# Evolution and Functional Diversification of the GLI Family of Transcription Factors in Vertebrates

**DOI:** 10.4137/ebo.s2322

**Published:** 2009-05-18

**Authors:** Amir Ali Abbasi, Debbie K. Goode, Saneela Amir, Karl-Heinz Grzeschik

**Affiliations:** 1 National Center for Bioinformatics, Faculty of Biological Sciences, Quaid-i-Azam University, Islamabad, 45320, Pakistan; 2 Institute of Human Genetics, Philipps-University, Bahnhofstrasse 7 D35037 Marburg, Germany; 3 School of Biological and Chemical Sciences, Queen Mary University of London, Mile End Road, London E1 4NS, U.K; 4 COMSATS Institute of Information Technology, Abbottabad, 22060, Pakistan

## Abstract

**Background:**

In vertebrates the “SONIC HEDGEHOG” signalling pathway has been implicated in cell-fate determination, proliferation and the patterning of many different cell types and organs. As the GLI family members (GLI1, GLI2 and GLI3) are key mediators of hedgehog morphogenetic signals, over the past couple of decades they have been extensively scrutinized by genetic, molecular and biochemical means. Thus, a great deal of information is currently available about the functional aspects of GLI proteins in various vertebrate species. To address the roles of GLI genes in diversifying the repertoire of the Hh signalling and deploying them for the vertebrate specifications, in this study we have examined the evolutionary patterns of vertebrate GLI sequences within and between species.

**Results:**

Phylogenetic tree analysis suggests that the vertebrate GLI1, GLI2 and GLI3 genes diverged after the separation of urochordates from vertebrates and before the tetrapods-bony fishes split. Lineage specific duplication events were also detected. Estimation of mode and strength of selection acting on GLI orthologs demonstrated that all members of the GLI gene family experienced more relaxed selection in teleost fish than in the mammalian lineage. Furthermore, the GLI1 gene appeared to have been exposed to different functional constraints in fish and tetrapod lineages, whilst a similar level of functional constraints on GLI2 and GLI3 was suggested by comparable average non-synonymous (Ka) substitutions across the lineages. A relative rate test suggested that the majority of the paralogous copies of the GLI family analyzed evolved with similar evolutionary rates except GLI1 which evolved at a significantly faster rate than its paralogous counterparts in tetrapods.

**Conclusions:**

Our analysis shows that sequence evolutionary patterns of GLI family members are largely correlated with the reported similarities and differences in the functionality of GLI proteins within and between the various vertebrate species. We propose that duplication and divergence of GLI genes has increased in the complexity of vertebrate body plan by recruiting the hedgehog signalling for the novel developmental tasks.

## Introduction

The GLI regulatory proteins act downstream of the secreted hedgehog (Hh) signalling molecules and are known to play an important role in vertebrate embryonic patterning in regions such as the central nervous system, the anterior-posterior axis of the embryonic limb bud, craniofacial structures and the lungs. Whilst *Drosophila* possesses a single homologue of GLI (cubitus interruptus, Ci), vertebrates have three members, characterized by five tandem C2-H2 zinc fingers linked by a consensus histidine-cysteine linker sequence.^1^ The birth of three GLI family members (GLI1, GLI2, and GLI3) from a single Ci like ancestral gene has been attributed to small scale gene duplication events that might have occurred within the time window of vertebrates-urochordates and fish-tetrapod split.^2^,^3^

Evidence from *Drosophila* suggests that all the Hh signalling is transduced via Ci protein.^4^ In the absence of Hh signalling the cytoplasmic Ci protein is cleaved to generate an N-terminal form with repressor activity. Hh signalling blocks this cleavage and increases the concentration of full length activator form of Ci protein. Thus a single *Drosophila* Ci protein can work both as an activator or repressor of target genes, upon the differential regulation of Hh signalling.^5^ Like Ci, the Hh signalling dependent cleavage plays an important role in the post-translational regulation of the vertebrate GLI proteins. However the activator and repressor functions of ancestral Ci protein are not distributed evenly among the three vertebrate GLI paralogs. Functionally, the *Drosophila* Ci is more closely related to vertebrate GLI2 and GLI3.^6^,^7^ These two partially redundant genes^8^,^9^ can activate transcription and undergo proteolysis to generate repressors of transcription.^10^ In contrast, GLI1 cannot undergo posttranslational modification and functions primarily as an activator of Hh transcriptional response.^11^ Genetic and biochemical studies in human, mice and frog suggest that during development the three GLI proteins act in combinatorial manner that is context dependent and species specific.^12^ For example, GLI1 and GLI2 induce motor neurons in the frog spinal cord, whereas GLI3 represses this function, by contrast, GLI1 induces floor plate differentiation in the same species, whereas both GLI2 and GLI3 repress this function.^13^ In mice GLI1 is not required for development or tumorigenesis,^14^,^15^ but it is essential for tumor formation in frog embryo and human cancers.^16^,^17^ Genetic studies with frogs and mice further suggest the divergent roles of GLI proteins in the patterning of the neural tube and CNS. For instance, during frog development each of the GLI proteins is critical in the induction of all primary neurons: motor, sensory and interneurons,^16^ whereas loss of any single or all GLI proteins in mouse embryos does not abolish neural tube development.^18^ Whilst there are divergent roles of GLI1 and GLI2 between mouse and zebrafish during development, the role of GLI3 appears to be conserved.^19^,^20^

Although the general aspects of GLI functions are similar in different vertebrate species, there are some important differences both at inter and intra-specific level. From an evolutionary perspective the duplication and divergence of GLI paralogs has increased the complexity of response to Hh morphogenetic signals in vertebrates. This complexity might, in turn, have contributed towards the deployment of Hh signalling to those domains of developing embryos which are considered as vertebrate synapomorphies, for instance appendicular (limb/fin) and craniofacial structures. To gain insight into the functional constraints operating on GLI family members (within and between the species) following the duplication events, we conducted a molecular evolutionary study comparing the tetrapod and teleost lineages. We demonstrated that all members of the GLI gene family experienced more relaxed selection in teleost fish than in mammalian lineage. We also found that GLI1 genes have been exposed to different functional constraints in fish and tetrapod lineages, whereas the GLI2 and GLI3 sequences were subjected to a similar level of functional constraints across the lineages. Additionally, we utilized a relative rate test to show that in majority of the species analyzed the paralogous copies of the GLI family evolved with similar evolutionary rates except in tetrapods where GLI1 evolved at a significantly faster rate than GLI2 and GLI3. Together, these results demonstrate that the evolutionary patterns of GLI sequences are largely correlated with their interspecific and intraspecific functional similarities and differences, but also show that duplication and divergence of GLI genes had led to the recruitment of the Hh pathway for the novel developmental processes in vertebrates.

## Results and Discussion

### Phylogenetic analysis

**Figure 1:**
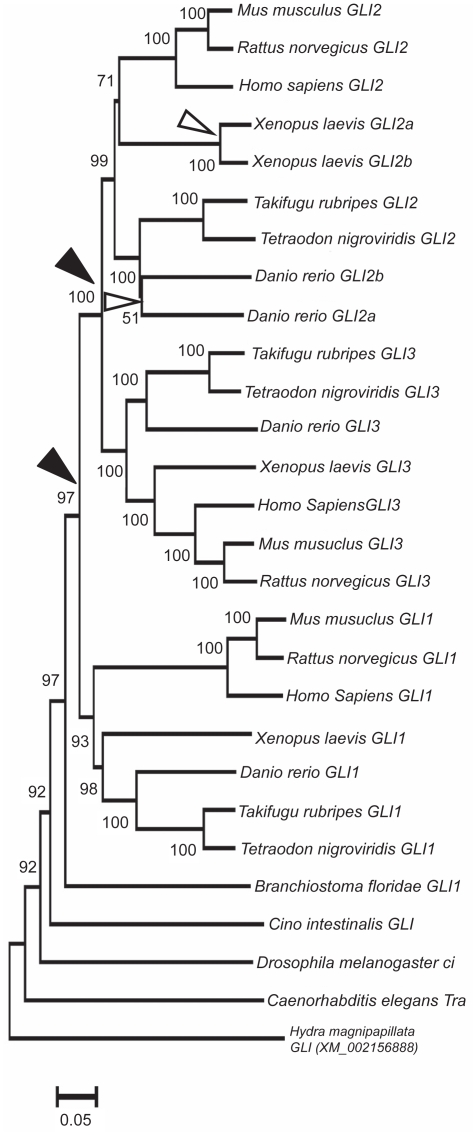
Neighbor-joining tree of the GLI family members. The percentage of replicate trees in which the associated taxa clustered together in the bootstrap test (1000 replicates) are shown next to the branches. The tree is drawn to scale, with branch lengths in the same units as those of the evolutionary distances used to infer the phylogenetic tree. All positions containing gaps and missing data were eliminated from the dataset. Black arrowheads show duplication events that occurred before the tetrapod-fish divergence whereas the open arrows indicate lineage specific duplications. Scale bar shows substitutions per site.

The phylogenetic history of vertebrate GLI genes was analyzed by including the sequences from representative members of teleost and tetrapod lineages (Fig. 1). The tree was rooted with orthologous genes from invertebrate species. A phylogenetic tree of multigene family members provides several types of useful information for studying the evolution and diversification of function of genes across various species. First, it can work as tool to provide support for or against direct orthologous relationships between genes from different species. Second, it can provide information on the likely status of members of gene family in animals that are ancestral to groups of currently extant species. Finally, the phylogenetic tree can provide an estimate of the relative time elapsed since the divergence of any two gene sequences from their most recent common ancestor.

With these points in mind the phylogenetic neighbor-joining (NJ) tree presented in Figure 1 reveals several interesting features of the vertebrate GLI gene family. The phylogeny shows a topology of the form (A) (BC) where vertebrate GLI2 and GLI3 genes cluster together with significant (99%) bootstrap support whereas GLI1 genes form an outgroup to them with bootstrap support of 100% (Fig. 1). The phylogeny suggests that, in the family of GLI genes, the ancestral chordate condition (as exemplified in the ciona/amphioxus) was likely a single, possibly *Amphioxus*-GLI like, copy of GLI gene.^21^ Then, before the actinopterygii-sarcopterygii split, the *Amphioxus*-GLI like ancestral gene underwent a duplication event and produced two gene copies, one of them (joint ancestor of GLI2 and GLI3) duplicated again, while other might not (GLI1) (Fig. 1). These three copies of an ancestral gene were then retained in both bony fishes and terrestrial vertebrates, because of their adoptive significance. The phylogeny further shows that GLI2 gene underwent lineage specific duplication events in zebrafish and *Xenopus* producing two gene copies independently in these two species (shown as GLI2a and GLI2b) (Fig. 1). Note that the branches of zebrafish GLI2a and GLI2b genes are long, suggesting that the duplication that gave rise to the extra copy of GLI2 gene in zebrafish is probably ancient, whereas the branch lengths of *Xenopus* GLI2a and GLI2b suggests that these genes arose relatively recently in the evolutionary history of this lineage.

### Estimation of sequence divergence among species

In order to determine the level of sequence divergence (influence of selection) at various phylogenetic separations, we sought to estimate the pattern of nucleotide substitutions at both silent (synonymous) and non-silent (non-synonymous) sites among GLI orthologs within and between the fish and tetrapod lineages. Selection was measured in terms of the difference in the rate of non-synonymous substitutions (Ka) to the rate of synonymous substitutions (Ks). If Ka and Ks values are not significantly different from each other this indicates that genes are under few or no selective constraints and thus evolving neutrally. The gene pair is said to be under negative selection, if the Ka value is significantly lower than Ks (Ka < Ks), i.e. non-silent substitutions have been purged by natural selection. The smaller the value of Ka compared to Ks, the larger the number of eliminated substitutions. The converse scenario, where the Ka value is significantly greater than Ks (Ka > Ks), is indicative of positive selection, i.e. advantageous mutations have accumulated during the course of evolution.

Ka and Ks values have been estimated in pairwise comparisons of orthologs using the Li-Wu-Lu method.^22^ Only those codons shared among all species have been considered for the analysis using the complete deletion option.

#### GLI3

Within the mammalian lineage the Ks values for the *GLI3* gene (Table 1) range from 0.051 (mouse-rat pair-wise comparison) to 0.194 (human-rat). Within the fish lineages the upper level of Ks substitutions approaches saturation level, i.e. Ks > 0.4 for zebrafish and tetraodon/*Fugu* comparisons. When using pair-wise comparisons between members of mammalian and fish lineages, both Ks and Ka values for GLI3 are in the range of 0.4–0.5.

#### GLI2

For the GLI2 gene (Table 1), Ks values within the mammalian lineage were similar to GLI3, whilst in fish lineages the upper limit of synonymous substitutions at Ks < 0.3 did not approach saturation. Mammalian-fish comparisons indicated a lower frequency of synonymous substitutions (0.271–0.368) compared to non-synonymous substitutions.

#### GLI1

Within both the mammalian and fish lineages the GLI1 Ks and corresponding Ka values are lower (Table 1), whilst between the two lineages the Ks values approached saturation (0.745–0.858). Corresponding non-synonymous substitution values (0.867–0.947) are higher than for GLI3 and GLI2 in pair-wise comparisons.

### Estimation of functional constraints

In order to estimate the selective forces operating on GLI gene family members following the duplication events, average Ka and Ks values have been estimated for GLI1, GLI2 and GLI3 genes, both within and between mammalian and fish lineages (Table 2). The t-value of difference between average Ka and Ks for each gene has then been used to estimate the significance to which they differ within and between mammalian and fish lineages. Results shown in Table 2 suggest that, with the exception of the mammalian-fish GLI2 comparison, there was no significant difference between the average Ka and Ks within or between the two. This indicates a strong trend towards neutrality (Ka/Ks ratio of 1) for substitution rates at synonymous and non-synonymous sites for GLI gene family. Only the mammalian-fish comparison for GLI2 suggests positive selection at 5% significance level (T = 2.43, p < 0.05).

Inspection of average Ka and Ks values (Table 2) revealed three important aspects of GLI evolutionary patterns. Firstly, all the three GLI gene family members showed a significantly higher rate of both silent and non-silent substitutions in fish when compared to mammals, suggesting a relatively relaxed selection in the fish lineage. This pattern correlates with the observations made by Robinson-Rechavi and Laudet^23^ who found that genes evolve faster in fish than in mammals. Secondly, between mammalian-fish lineages, the significantly higher average Ka and Ks values for GLI1 compared to GLI2 and GLI3 indicates relaxed selection and accelerated evolution in GLI1. This is perhaps reflected in the divergent GLI1 functions attained in teleosts and tetrapods^19^ since they last shared a common ancestor 450 million years ago. Thirdly, between mammalian-fish GLI2 and GLI3 genes, not only the average Ka values, (usually subject to selective pressure) but also the corresponding Ks (assumed to be neutral) values are significantly lower than saturation level (Ks > 5) (Table 2). This indicates that strong purifying selection operates on both silent and non-silent sites. The lower rate of substitutions at silent sites is suggestive of codon usage bias in these two genes.^24^,^25^ Furthermore average Ka values for GLI2 and GLI3 between mammalian-fish lineages are similar, perhaps due to equivalent functional constraints imposed on both genes.

Whilst GLI1 appears to have undergone rapid evolution since the divergence of tetrapods and teleosts, the GLI2 and GLI3 sequences appear to have evolved at considerably slower rate. This data is consistent with the functional conservation of GLI3 in vertebrates,^20^ but not with experi men tal data that indicates a functional divergence of GLI2 orthologs in mice and zebrafish.^19^ This functional divergence of GLI2 can be explained by two scenarios, by accommodating subtle changes (non-silent) within critical functional domains of the protein in each lineage, leading to functional divergence or perhaps by changes in gene expression pattern while keeping the protein activity domains conserved throughout the course of evolution.

### Evolutionary distance between paralogs

To determine the evolutionary rates with which the duplicated genes evolved in each species tested (human, mouse, rat, frog, *Fugu*, teraodon, zebrafish) the Tajima relative rate test^26^ has been carried out (Table 3) on amino acid substitutions on pairs of GLI paralogs, by using the orthologous sequence Ci from *Drosophila* as an outgroup. The Tajima relative rate test determines whether one duplicate has diverged to a greater extant than the other by comparing the sequences of each of the paralogs with that of the ortholog used as the outgroup. The results of this analysis (Table 3) indicate that in most cases (16/21 pairs) the GLI paralogs evolved at similar rate in each animal analyzed. Our findings in the relative rate test are in agreement with Hughes and Hughes^27^ and Kondrashov et al.^28^ who suggested that paralogs typically evolve at similar rates, without significant asymmetry.

The markedly increased evolutionary rate (p < 0.05) of GLI1 in human and mouse may reflect profound changes in the function of this gene compared to either of its paralogs in mammals. This notion is compatible with results from functional studies, where GLI2 and GLI3 are found to perform overlapping activities in mammalian cell culture and transgenic experiments, while GLI1 appears to play a notably different role.^10^,^11^ Faster evolutionary rate also suggests that orthologous copies of GLI1 gene in human and mice might have attained divergent roles during the course of evolution. This assumption is in harmony with the functional data which shows that in mice GLI1 is not required for development or tumorigenesis, but it is essential for the proliferation of human tumor cells.^15^,^17^ Asymmetric evolution of frog GLI paralogs probably suggests a trend in tetrapod GLI1 gene to experience an increased evolutionary rate (under relaxed selection pressure), whereas rapid evolution of GLI2 (evolutionary rate is comparable to GLI1 paralog, Table 3) might indicate the functional redundancy of GLI2 duplicates (GLI2a and GLI2b) in amphibians.

## Conclusions

The Hh signalling pathway first elucidated in *Drosophila* and subsequently the vertebrate homologs of *Drosophila* Hh pathway genes were described by genetic studies in mouse, frog and zebrafish. These studies demonstrated that Hh signalling in vertebrates shares many features with that in insects, although clear differences have emerged. For instance, many genes involved in this pathway expanded by gene duplication specifically in vertebrate lineage. GLI proteins act at the last known step of Hh signalling pathway and lead to the activation or repression of target genes in a cellular context dependent manner. The fact that vertebrates possess more copies of GLI genes than did the common ancestor of chordates, suggests that the duplication and divergence of GLI genes in vertebrates has diversified the mechanisms of receiving and interpreting the Hh signalling. This increase in the genetic complexity of Hh pathway mediators in early vertebrate evolution could conceivably be one of the key factors underlying the evolution of vertebrate innovations, including the limbs, bone and craniofacial structures. In this study we have inspected the molecular evolution of GLI family members in vertebrates. All the three GLI genes show a higher degree of divergence at both synonymous and non-synonymous sites in the teleost lineage when compared to mammals. This difference may indicate that GLI orthologs have achieved a greater level of functional diversification in the fish lineage. In mammalian-fish sequence comparisons it appeared that GLI1 have accumulated significantly more synonymous and non-synonymous changes than GLI2 and GLI3. This may reflect functional importance associated with evolutionary pressure to retain the sequence features of two copies of the GLI family across the vertebrate lineage, whereas the third copy was free from constraining effects of natural selection and has attained unique features in each lineage. The findings from a relative rate test involving GLI paralogs from each species examined suggest that the GLI1 protein may have undergone an accelerated evolutionary rate not only at interspecific level but also at intraspecific level. We propose that a transition from a single, *Amphioxus-*GLI like, ancestral chordate gene to three distinct vertebrate GLIs and their subsequent interspecific and intraspecific diversifications were critical events in diversifying the repertoire of the Hh signalling and deploying them for the vertebrate specifications.

## Materials and Methods

In order to analyze the evolutionary patterns/history of GLI sequences the complete cDNAs and corresponding protein sequences for human GLI gene family members, i.e. GLI1, GLI2 and GLI3 and their orthologs in mouse, rat, frog, *Fugu*, tetraodon, zebrafish, and several invertebrate species (Table 4) were extracted from ENSEMBL genome browser (http://www.ensembl.org) and National Center for Biotechnology Information (http://www.ncbi.nlm.nih.gov).

The phylogenetic tree for the GLI gene family was reconstructed by using the neighbor-joining method.^29^,^30^ All positions containing gaps and missing data were eliminated from the dataset. Reliability of the resulting tree topology was tested by the bootstrap method (at 1000 pseudoreplicates) which generated the bootstrap probability for each interior branch in the tree.^31^ The phylogenetic tree was rooted with orthologous genes from invertebrates.

Number of synonymous nucleotide substitutions per synonymous (Ks) and non-synonymous nucleotide substitutions per non-synonymous site (Ka) were calculated by using the Li-Wu-Lu method^22^ in pairwise comparison.

Evolutionary distance between all possible pairs of GLI paralogs within each lineage was estimated by Tajima’s relative rate test.^26^

## Figures and Tables

**Table 1:** Estimation of Ks and Ka values in pair-wise comparisons.

	Human	Mouse	Rat	Tetraodon	Fugu	Zebrafish
**GLI3**						
Human		0.170 (0.013)	0.185 (0.013)	0.646 (0.027)	0.480 (0.026)	0.487 (0.026)
Mouse	0.161 (0.022)		0.060 (0.007)	0.466 (0.026)	0.491 (0.026)	0.467 (0.025)
Rat	0.194 (0.024)	0.051 (0.011)		0.485 (0.028)	0.507 (0.028)	0.462 (0.025)
Tetraodon	0.426 (0.040)	0.494 (0.046)	0.485 (0.043)		0.106 (0.010)	0.426 (0.024)
Fugu	0.449 (0.043)	0.449 (0.043)	0.507 (0.043)	0.106 (0.015)		0.427 (0.025)
Zebrafish	0.489 (0.043)	0.486 (0.044)	0.462 (0.045)	0.426 (0.040)	0.427 (0.041)	
**GLI2**						
Human		0.154 (0.013)	0.144 (0.013)	0.477 (0.029)	0.451 (0.029)	0.450 (0.030)
Mouse	0.115 (0.018)		0.068 (0.008)	0.541 (0.030)	0.479 (0.031)	0.462 (0.029)
Rat	0.112 (0.018)	0.032 (0.010)		0.489 (0.029)	0.465 (0.030)	0.470 (0.030)
Tetraodon	0.330 (0.035)	0.365 (0.039)	0.368 (0.039)		0.170 (0.015)	0.422 (0.026)
Fugu	0.271 (0.031)	0.330 (0.036)	0.337 (0.036)	0.111 (0.018)		0.403 (0.025)
Zebrafish	0.306 (0.034)	0.334 (0.037)	0.322 (0.036)	0.296 (0.034)	0.227 (0.028)	
**GLI1**						
Human		0.160 (0.010)	0.166 (0.011)	0.887 (0.042)	0.871 (0.041)	0.924 (0.046)
Mouse	0.215 (0.021)		0.059 (0.006)	0.906 (0.044)	0.889 (0.042)	0.947 (0.048)
Rat	0.212 (0.021)	0.074 (0.021)		0.901 (0.045)	0.867 (0.041)	0.904 (0.045)
Tetraodon	0.799 (0.059)	0.837 (0.063)	0.828 (0.063)		0.091 (0.007)	0.380 (0.019)
Fugu	0.793 (0.059)	0.868 (0.065)	0.858 (0.068)	0.139 (0.016)		0.366 (0.017)
Zebrafish	0.745 (0.053)	0.788 (0.057)	0.765 (0.056)	0.386 (0.031)	0.377 (0.032)	

The first column and row gives the name of species for which the pair-wise comparisons were performed. For each member of the GLI gene family (first column) the numbers of synonymous substitutions per synonymous site (Ks) and numbers of non-synonymous substitutions per non-synonymous site (Ka) are, respectively, presented below and above the diagonal.

**Table 2:** Average Ka and Ks values between and within mammalian-fish lineages for GLI orthologs.

	Ka	Ks	t-value of difference	Doublesided p-value	Difference between Means (Ka − Ks)
**GLI1**					
Mammals-Fish	0.621 ± 0.023 (0.361)	0.579 ± 0.032 (0.371)	0.314	0.7556	non-significant
Mammals	0.130 ± 0.007 (0.056)	0.177 ± 0.014 (0.109)	−0.664	0.5532	non-significant
Fish	0.299 ± 0.011 (0.159)	0.348 ± 0.023 (0.139)	−0.402	0.8013	non-significant
**GLI2**					
Mammals-Fish	0.374 ± 0.021 (0.152)	0.257 ± 0.021 (0.108)	2.43	0.0206	significant
Mammals	0.133 ± 0.009 (0.054)	0.129 ± 0.015 (0.066)	0.081	0.9586	non-significant
Fish	0.339 ± 0.021 (0.176)	0.341 ± 0.020 (0.178)	−0.014	0.9931	non-significant
**GLI3**					
Mammals-Fish	0.379 ± 0.025 (0157)	0.372 ± 0.015 (0.162)	0.12	0.9052	non-significant
Mammals	0.155 ± 0.025 (0.070)	0.161 ± 0.025 (0.080)	−0.098	0.9507	non-significant
Fish	0.260 ± 0.011 (0.146)	0.421 ± 0.027 (0.261)	−0.935	0.4285	non-significant

t and p values of pair-wise t-tests are also indicated. ± sign represents standard errors, and standard deviations are enclosed within the brackets.

**Table 3:** Tajima’s relative rate test for the comparison of evolutionary distance between GLI paralogs in different species using the *Drosophila* Ci as an outgroup.

Evolutionary Distance	x^2^	df	p
**Human**			
GLI1 vs GLI2	6.43	1	0.011*
GLI1 vs GLI3	5.24	1	0.022*
GLI2 vs GLI3	0.20	1	0.652
**Mouse**			
GLI1 vs GLI2	9.19	1	0.002*
GLI1 vs GLI3	7.01	1	0.008*
GLI2 vs GLI3	0.17	1	0.676
**Rat**			
GLI1 vs GLI2	3.33	1	0.068
GLI1 vs GLI3	3.57	1	0.059
GLI2 vs GLI3	0.54	1	0.463
**Frog**			
GLI1 vs GLI2	0.30	1	0.581
GLI1 vs GLI3	3.21	1	0.073
GLI2 vs GLI3	5.80	1	0.016*
**Tetraodon**			
GLI1 vs GLI2	3.42	1	0.064
GLI1 vs GLI3	2.14	1	0.143
GLI2 vs GLI3	0.31	1	0.579
**Zebrafish**			
GLI1 vs GLI2	1.03	1	0.310
GLI1 vs GLI3	0.17	1	0.680
GLI2 vs GLI3	0.01	1	0.920
**Fugu**			
GLI1 vs GLI2	1.27	1	0.259
GLI1 vs GLI3	0.32	1	0.574
GLI1 vs GLI3	2.04	1	0.153

P-value with “*” symbol represents the situation where GLI1 (human and mouse) and GLI2 (frog) evolves significantly (p < 0.05) faster than the counterpart.

**Table 4:** ENSEMBL and NCBI derived Peptides and cDNAs used to analyze the sequence evolutionary patterns of GLI genes.

Sequence	Peptide ID	Transcript ID
**Human**		
GLI1	ENSP_228682	ENST_228682
GLI2	ENSP_354586	ENST_361492
GLI3	ENSP_265526	ENST_265526
**Mouse**		
GLI1	ENSMUSP_26474	ENSMUST_26474
GLI2	ENSMUSP_70591	ENSMUST_63361
GLI3	ENSMUSP 21754	ENSMUST 21754
**Rat**		
GLI1	ENSRNOP_9803	ENSRNOT_9803
GLI2	ENSRNOP_9963	ENSRNOT_9963
GLI3	ENSRNOP_19396	ENSRNOT_19396
**Frog**		
GLI1	Q91690	U57454
GLI2a	NP_001081894	NM_001088425
GLI2b	NP_001081442	NM_001087973
GLI3	NP_001081440	NM_001087971
**Fugu**		
GLI1	NEWSINFRUG_154410	NEWSINFRUT_164302
GLI2	NEWSINFRUP_159280	NEWSINFRUT_159280
GLI3	NEWSINFRUP_163565	NEWSINFRUT_163565
**Tetraodon**		
GLI1	GSTENT_13570001	GSTENT_13570001
GLI2	GSTENP_33101001	GSTENT_33101001
GLI3	GSTENP_25555001	GSTENT_25555001
**Zebrafish**		
GLI1	NP_840081	NM_178296
GLI2a	NP_571042	NM_130967
GLI2b	NP_001015069	NM_001015069
GLI3	NP_991291	NM_205728
**Amphioxus**		
GLI	CAB96572	AJ252244
**Ciona**		
GLI	XP_002120619	XM_002120583
***Drosophila***		
Ci	CG2125-PA	CG2125-RA
***C. elegans***		
Tra	NP_001022881	NM_001027710
***Hydra***		
GLI	XP_002156924	XM_002156888
